# Global Methylation and Hydroxymethylation in DNA from Blood and Saliva in Healthy Volunteers

**DOI:** 10.1155/2015/845041

**Published:** 2015-05-18

**Authors:** Lode Godderis, Caroline Schouteden, Ali Tabish, Katrien Poels, Peter Hoet, Andrea A. Baccarelli, Kirsten Van Landuyt

**Affiliations:** ^1^Centre Environment & Health, Department of Public Health and Primary Care, KU Leuven, Kapucijnenvoer 35/5, 3000 Leuven, Belgium; ^2^IDEWE, External Service for Prevention and Protection at Work, 3001 Heverlee, Belgium; ^3^Department of Environmental Health, Landmark Center, Suite 415 West, P.O. Box 15677, 401 Park Drive, Boston, MA 02215, USA; ^4^Department of Oral Health Sciences, KU Leuven BIOMAT, 3000 Leuven, Belgium

## Abstract

*Aims*. We describe a liquid chromatography-tandem mass spectrometry (LC-MS/MS) method to quantify and compare simultaneously global methylation and hydroxymethylation in human DNA of different tissues. *Materials and Methods*. Blood and saliva DNA from fourteen volunteers was processed for epigenetic endpoints using LC-MS/MS and PCR-pyrosequencing technology. *Results*. Global DNA methylation was significantly lower in saliva (mean 4.61% ±  0.80%), compared to blood samples (5.70% ± 0.22%). In contrast, saliva (0.036% ± 0.011%) revealed significantly higher hydroxymethylation compared to blood samples (mean 0.027% ± 0.004%). Whereas we did not find significant correlations for both epigenetic measures between the tissues, a significant association was observed between global methylation and global hydroxymethylation in saliva DNA. Neither LINE-1 nor Alu elements of blood and saliva correlated, nor were they correlated with the DNA hydroxymethylation of blood or saliva, respectively. *Conclusion*. Global DNA methylation and hydroxymethylation of cytosine can be quantified simultaneously by LC-MS/MS. Saliva DNA cannot be considered as a surrogate for blood DNA to study epigenetic endpoints.

## 1. Introduction

Epigenetics refers to the study of changes in gene function that are mitotically and/or meiotically heritable and that do not entail a change in DNA sequence [1]. Epigenetic mechanisms are essential for development (genome imprinting, X chromosome inactivation, etc.) and differentiation (transcriptional regulation) but can be disrupted by exogenous agents [2]. Epigenetic changes have been described in relation to environmental exposure similar to changes observed in chronic diseases, such as cancer or Alzheimer disease. The most investigated epigenetic mechanisms include DNA methylation, histone modifications, and RNA-mediated silencing [3, 4].

DNA cytosine methylation (5mC) is chemically relatively stable, but still dynamic epigenetic modifications regulated by DNA methyltransferases (DNMTs) often occur. These alterations involve the covalent addition of a methyl group to the 5-position of cytosine with S-adenosyl methionine (SAM) as the methyl donor [5, 6]. DNA methylation is almost exclusively restricted to CpG dinucleotides clustered within the gene promoter and in repeated elements such as long (LINE-1) and short (Alu) interspersed elements [5, 7]. Paradoxically, 90% of the methylated CpG lies outside the coding regions such as CpA, CpT, and CpNpG sites, possibly to serve as repressors of transposons or viral-like transcripts [8]. In general, hypermethylation of the DNA promoter regions inactivates the gene expression and hypomethylation activates the expression [9]. Aberrant 5mC levels, that is, global hypomethylation and/or gene-specific hypermethylation or hypomethylation, are observed in diseases like leukemia and cancer but have also been observed in cells exposed to carcinogenic agents [10–12].

Besides 5mC, other epigenetic modifications, such as 5-hydroxymethylcytosine (5hmC), have been recently discovered in this rapidly evolving field. Significant levels of 5hmC have been found in DNA from embryonic stem cells, neurons, and brain [13]. Even though the biological function is not yet completely clarified, 5hmC is of special interest in order to understand the regulation of gene expression and chromatin structure since it acts as an oxidized intermediate in the active demethylation of 5mC or even may be the final product of genome-wide demethylation [13, 14].

The conversion of 5mC to 5hmC is catalysed by ten eleven translocation (TET) family of proteins (TET1, TET2, and TET3) which are 2-oxoglutarate (2OG-) and Fe(II)-dependent dioxygenases. TET1 and TET2 are involved in the maintenance of embryonic stem cells pluripotency and cell lineage commitment. TET1 is a fusion partner of the MLL gene in rare cases of acute myeloid and lymphoid leukemias. TET2 modulates the balance between self-renewal and differentiation in hematopoietic stem cells, making them critical for normal myelopoiesis. Loss-of-function of TET2 is associated with acute myeloid leukemia, myelodysplastic and myeloproliferative disorders. TET3 contributes to the global DNA demethylation during the zygotic stage of embryonic development [14]. These studies highlight the role of TET-mediated 5-hmC in the developmental processes and the possibility that altered DNA 5hmC levels can lead to malignancy [5, 14].

Beside the TET pathway, 5hmC can also be formed by other mechanisms, for example, UV irradiation of 5mC in aerated aqueous solution and DNA methyltransferase reaction of cytosine with formaldehyde. The process of active DNA demethylation via 5hmC also seems to be mediated by activation-induced deaminase and DNA glycosylase, which are involved in deaminating and excision repair [5]. This indicates that environmental factors might modify the DNA methylation and hydroxymethylation status and that assays are needed to measure both total DNA methylation and hydroxymethylation [15].

*In vivo* studies on the epigenetic effects of environmental carcinogens are scarce and have mainly been performed on human lymphocytes [11, 12]. Blood samples are often the preferred source of genetic material because they provide large amounts of cells and in the same sample a wide range of environmental agents can be determined [16]. The desire for large-scale epidemiological studies involving thousands of participants necessitates less invasive and more cost-efficient procedures for collecting DNA that would facilitate the trial recruitment [17]. Saliva and buccal swab samples are described as a noninvasive alternative to collect human DNA for epigenetic epidemiological studies [18, 19]. Previous research showed that sufficient amounts of high-quality DNA could be collected from saliva [20]. The potential advantages of saliva sample collection compared with blood sample collection include lower overall cost, lower infection risk, increased patient convenience, acceptability, and compliance [21]. However, a potential drawback is the presence of exogenous DNA (e.g., from bacteria) commonly present in human saliva and buccal swab samples [22].

In this paper we report on the application of a fast and sensitive liquid chromatography-tandem mass spectrometry (LC-MS/MS) method for the simultaneous quantification of 5mC and 5hmC in human DNA from different tissues. We investigated the association between both epigenetic marks and compared the results with LINE-1 and Alu methylation, often used as surrogates for global DNA methylation in monitoring studies, determined with PCR-pyrosequencing. In addition, we also investigated for the first time whether these epigenetic endpoints in DNA of saliva are comparable with DNA from blood of human volunteers in order to serve as a noninvasive alternative for biomonitoring purposes.

## 2. Material and Methods

### 2.1. Study Design and Population

Fourteen healthy volunteers (*n* = 4 male, *n* = 10 female) aged less than 45 years were enrolled in this study. Most of the volunteers (*n* = 12) were Caucasian. The participants were recruited among the scientific staff of the Department of Oral Health Sciences and the Department of Public Health and Primary Care and among KU Leuven pregraduate medical students. All participants received information about the purpose and objectives of the study and gave written informed consent to the proposed processing of the data. Participants were asked to fill out a small questionnaire on general health and lifestyle. The study was approved by the Commission for Medical Ethics of UZ Leuven (reference number S53445).

### 2.2. Sample Collection

Donors were refrained from eating and drinking for at least 8 hours prior to sample collection and were asked to rinse their mouth prior to sample taking. Subjects were asked to produce 2 mL unstimulated saliva using the self-collection kit OG-500 from Oragene (DNA GenoTek, Ottawa, OT, Canada). Next, blood was drawn from each participant (three EDTA tubes of 4.5 mL).

### 2.3. DNA Extraction

DNA extraction was performed with GeneCatcher gDNA Blood Kit for blood samples and Oragene OG-500 kit (DNA GenoTek) for saliva samples. The quantity and purity of DNA were determined by a NanoDrop spectrophotometer.

### 2.4. DNA Methylation and DNA Hydroxymethylation Analysis

DNA was analyzed by LC-MS/MS as described previously [23]. Briefly, isolated genomic DNA samples (1 *μ*g) were enzymatically hydrolyzed to individual deoxyribonucleosides by a simple one-step DNA hydrolysis procedure. A digest mix was prepared by adding phosphodiesterase I, alkaline phosphatase, and Benzonase Nuclease to Tris-HCl buffer. Extracted DNA was hydrolyzed by adding 50 *μ*L digest mix and incubating at 37°C for at least 8 h. After hydrolysis, 900 *μ*L of HPLC-grade water was added to each sample. Exposure to daylight was avoided over the entire sample preparation procedure in order to minimize potential deamination of the target compounds.

Stock solutions of 5-methyl-2′-deoxycytidine (5mdC), 5-hydroxymethyl-2′-deoxycytidine (5hmdC), and 2′-deoxycytidine (dC) were prepared by dissolving commercial solid reference standards in HPLC-grade water. Stock solutions were used to prepare calibration standards. Global DNA methylation and hydroxymethylation were obtained by quantifying 5mdC, 5hmdC, and dC using ultrapressure liquid chromatography (UPLC), in combination with tandem mass spectrometry (MS-MS). LC/MS-MS analysis of the samples was conducted on a Waters Acquity UPLC, coupled to a Waters Micromass Quattro Premier Mass Spectrometer using electrospray ionization (ESI). A 15 *μ*L aliquot of the sample was introduced on an Acquity UPLC BEH C_18_, 50 mm × 2.1 mm, 1.7 *μ*m column, held at a temperature of 40°C. The mobile phase used for the chromatographic separation was a mixture of 0.1% formic acid in water (A) and 0.1% formic acid in acetonitrile (B) using the following gradient: the program was starting at 10% B, increasing linearly to 100% B for 2 min, then held from 2 to 2.1 min at 100% B, and finally brought back to the initial status from 2.1 to 3.0 min. A flow rate of 0.35 mL/min was applied. The analyses were performed in the positive ESI mode and a multiple reaction monitoring (MRM) method was used with argon as the collision gas.

All DNA samples isolated from blood and saliva were hydrolysed in triplicate. Each sample was then analysed twice using the LC-MS/MS method. By interpolation from the established calibration curves, the absolute concentrations, expressed in ng/mL, for 5mdC, dC, and 5hmdC, present in the samples (1 *μ*g DNA/mL), could be derived. Together with every set of 14 volunteer DNA samples, also 3 quality control (QC) DNA samples have been prepared and analysed, in order to uncover any potential errors upon sample preparation and analysis. Global DNA methylation is expressed as a percentage of 5mdC versus the sum of 5mdC, 5hmdC, and dC [%Methylation = 5mdC/(5mdC + 5hmdC + dC)], while global DNA hydroxymethylation is expressed as a percentage of 5hmdC versus the sum of 5mdC, 5hmdC, and dC [%Hydroxymethylation = 5hmdC/(5mdC + 5hmdC + dC)].

### 2.5. Pyrosequencing of LINE-1 and Alu Elements

Long interspersed nucleotide elements (LINE-1) and the AluSX (Alu) methylation levels were assessed using PCR-pyrosequencing of bisulfite-treated DNA. Details of the PCR-pyrosequencing assays used in the current study are described by Kile, 2010 [7].

### 2.6. Statistical Analysis

Differences between groups were analysed by Mann Whitney *U* test for unrelated data and Wilcoxon signed rank test for related data. Within-tissue and between-tissue DNA association of methylation and hydroxymethylation levels was investigated by spearman correlation.

## 3. Results

### 3.1. Characteristics of Participants and Method

A total of 14 healthy subjects (4 males, 10 females) enrolled in the study. Mean age was 29 years (range 22–43) and BMI ranged between 20 and 29 (mean 24). Six subjects reported a history of allergy of dust mites, hay fever, asthma, eczema, penicillin, or nickel allergy.

The Oragene saliva collection kit yielded sufficient DNA out of 2 mL saliva for all participants with a mean yield of 20.1 *μ*g DNA (range 6.8–135.4 *μ*g DNA). To perform LC-MS analysis, a minimum concentration of 20 ng DNA/*μ*L is required. We extracted on average 42.4 *μ*g DNA (range 1.8–97.4 *μ*g) out of 4.5 mL blood. The minimum 260/280 ratio was >1.6 for DNA of both tissues.

**Figure 1:**
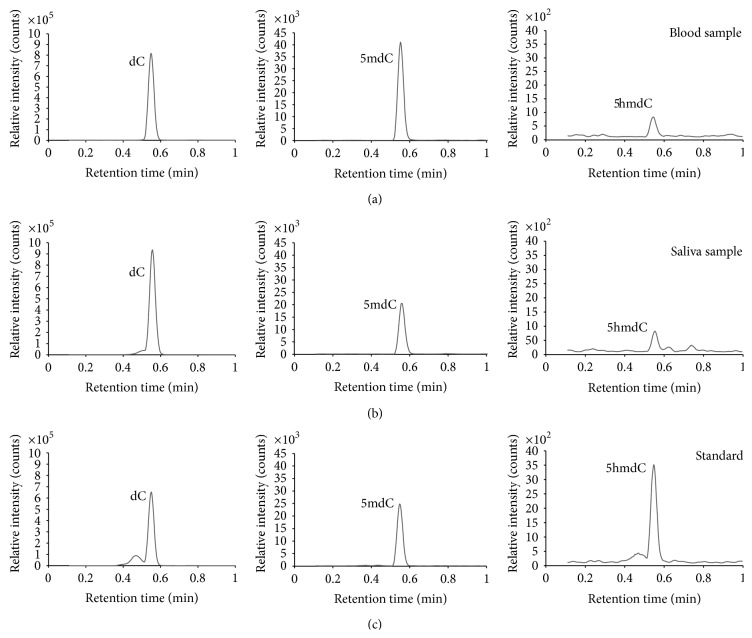
UPLC chromatograms of the monitored ion transitions for 2′-deoxycytidine (dC), 5-methyl-2′-deoxycytidine (5mdC), and 5-hydroxymethyl-2′-deoxycytidine (5hmdC): (a) blood sample from a volunteer, (b) saliva sample from the same volunteer, and (c) calibration standard, containing dC (30.7 ng/mL), 5mdC (1.5 ng/mL), and 5hmdC (0.07 ng/mL).

**Figure 2:**
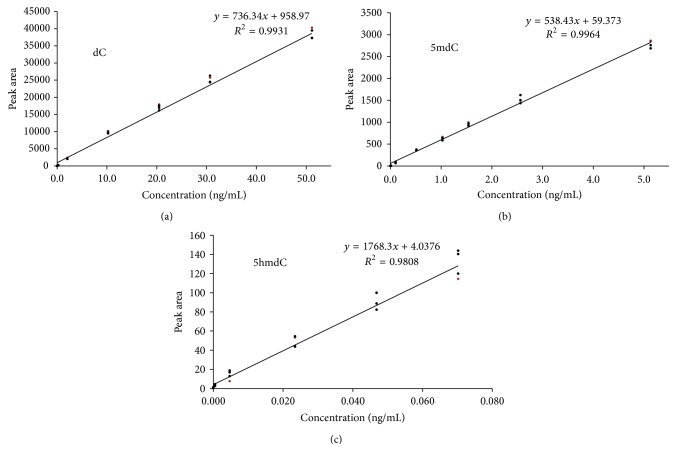
Calibration curves for (a) 2′-deoxycytidine (dC), (b) 5-methyl-2′-deoxycytidine (5mdC), and (c) 5-hydroxymethyl-2′-deoxycytidine (5hmdC).

In order to perform the DNA (hydroxy)methylation analysis of the samples, a calibration series in HPLC-grade water was prepared for 5mdC, dC, and 5hmdC and run by the analytical method. Calibration standards were prepared, starting from purchased reference standards, in a range of, respectively, 0.01–5 ng/mL for 5mdC, 0.2–50 ng/mL for dC, and 0.005–0.07 ng/mL for 5hmdC. The same calibration standards were used in all experiments. Upon LC/MS-MS analysis, independent quantification of 5mdC, dC, and 5hmdC was possible since different, unique transitions from precursor ion to product ion were monitored in MRM mode:* m*/*z* 242.2 → 125.95 for 5mdC (cone voltage 20 V, collision energy 14 eV),* m*/*z* 228.1 → 111.9 for dC (cone voltage 18 V, collision energy 12 eV), and* m*/*z* 258.0 → 141.9 for 5hmdC (cone voltage 15 V, collision energy 10 eV). The dwell time per transition was 90 ms. Calibrations solutions were analysed in MRM mode and data processing was based on absolute peak areas of the different unique product ions. In Figure 1, the UPLC chromatograms are shown for dC, 5mdC, and 5hmdC, for a volunteer's blood and saliva sample, as well as for a calibration standard. For the three different compounds, calibration curves were constructed, as presented in Figure 2.

For 5mdC and dC the established linear calibration curves had correlation coefficients >0.99 while for 5hmdC a correlation coefficient around 0.98 was observed (see Figure 2). It should be noted however that the concentration range for 5hmdC is situated far below the ones for 5mdC and dC. Using an amount of 1 *μ*g digested DNA, the method's limits of detection (LODs) for 5 mdC, dC, and 5hmdC were, respectively, 0.01 ng/mL, 0.01 ng/mL, and 0.005 ng/mL. As a sample aliquot of 15 *μ*L is injected, these LODs correspond to amounts of 0.62 fmol 5mdC, 0.66 fmol dC, and 0.29 fmol 5hmdC injected on-column. These LODs are comparable to previously LC/MS-MS sensitivity data, reported by Thuc et al. (0.5 fmol for 5mdC and 5hmdC) [24].

### 3.2. Results of %Methylation and %Hydroxymethylation

In Table 1 the results of %DNA methylation and %DNA hydroxymethylation are presented. The mean value of global DNA methylation was significantly (*P* = 0.001) lower in saliva samples (mean 4.61%), compared to blood samples (mean 5.70%). This is in contrast with global hydroxymethylation, which was significantly higher (*P* = 0.001) in saliva samples (mean 0.036%), compared to blood samples (mean 0.027%).

We did not observe significant differences in global DNA methylation and hydroxymethylation levels between males and females. In contrast, 6 individuals with allergy (0.029% ± 0.002%, 0.027%–0.032%) showed a small but significant increase (*P* = 0.042) in %hydroxymethylation in DNA from blood compared to 8 nonallergic participants (0.025% ± 0.004%; 0.021%–0.031%). Neither age nor BMI seemed to influence the epigenetic endpoints; however only subjects under 45 were allowed to participate.

Next, we compared %DNA methylation in blood and saliva and %DNA hydroxymethylation in blood and saliva. No significant association could be observed (spearman rho = 0.141, *P* = 0.631 and −0.021, *P* = 0.943) for both epigenetic endpoints between the two tissues.

**Figure 3:**
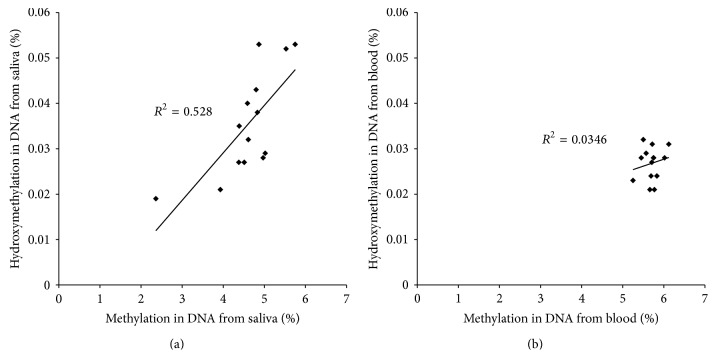
Association between %DNA methylation and %DNA hydroxymethylation in (a) saliva samples and (b) blood samples.

We also performed the correlation between %DNA methylation and %DNA hydroxymethylation in the same tissue. A significant association was found between %methylation and %hydroxymethylation in saliva DNA (spearman rho = 0.716; *P* = 0.004) (Figure 3(a)). In blood, no such significant correlations could be revealed (spearman rho = 0.056; *P* = 0.850) (Figure 3(b)).

### 3.3. LINE1 and Alu Methylation

It is suggested that analyzing the methylation of DNA repetitive elements can serve as a surrogate marker for global genomic DNA methylation. We thus assessed the methylation levels of LINE-1 and Alu elements of blood and saliva DNA (see Supplementary  Table  1 available online at http://dx.doi.org/10.1155/2014/845041). No significant correlation was observed between the methylation levels of blood and saliva Alu elements (spearman rho = 0.297, *P* = 0.303). Methylation levels of blood and saliva LINE1 elements were also not significantly correlated (spearman rho = −0.196, *P* = 0.503), although in general lower methylation levels were observed for LINE1 and Alu elements in saliva DNA compared to their methylation levels in blood DNA (Supplementary Table  1). Also, no significant correlations were observed in the methylation levels of Alu (spearman rho = −0.117, *P* = 0.690) and LINE elements (spearman rho = 0.155, *P* = 0.598) of saliva with the DNA hydroxymethylation in saliva and in the methylation levels of Alu (spearman rho = 0.139, *P* = 0.635) and LINE1 elements (spearman rho = 0.099, *P* = 0.737) of blood with the DNA hydroxymethylation level of blood.

## 4. Discussion

We described the method for simultaneous quantification of global DNA methylation and hydroxymethylation by LC-MS/MS with a high sensitivity and accuracy. The DNA methylation pattern is in line with a previously published study [25]. By applying the calibrated assay we showed that 5hmC is present in DNA of different human tissues. Little is known about the global 5hmC levels in different species. We report lower global 5hmC contents compared to the other studies reporting global DNA 5hmC contents in different nonhuman tissues. Different detection and expression methods for 5hmC used in small number of studies investigating the global DNA 5hmC contents make it difficult to compare them with our findings of global DNA 5hmC levels in blood and saliva.

Global DNA methylation levels in males and females were comparable, which is in line with published data [26]. Gender also does not seem to affect global hydroxymethylation levels in DNA from blood or saliva. Since we limited the age at participation, we cannot draw conclusion on the absence of an age-effect on global DNA hydroxymethylation. The effect of age on global DNA methylation has been widely discussed [27]. Several studies based on relatively small study samples (between 76 and 237 subjects) reported an inverse association between age and genomic 5mC content from blood of healthy subjects [27]. In contrast, other studies of similar size or larger (between 32 and 526 samples) reported no association of age with genomic 5mC content [28].

One of theobjectives was to determine whether saliva could be a reliable source of DNA and serve as a noninvasive alternative of blood DNA in biomonitoring studies. Sufficient good quality DNA could be extracted from saliva and both quality and quantity were in line with previously published data [16, 17, 29]. We did not observe any association between the two tissues for both epigenetic endpoints (global DNA methylation and DNA hydroxymethylation), and also no correlation was observed between the LINE1 and Alu elements of both tissues. On the one hand, this could be due to the lack of power due to the small sample size or due to differences in DNA extraction methods [29].

On the other hand, it is known that saliva samples are contaminated with DNA from oral bacteria and/or food, which can overestimate the amount of DNA in these samples [16, 17, 21]. In our study, participants were refrained from eating and drinking 8 hours prior to the sample collection and mouth was rinsed prior to sampling. In addition, the Oragene sample kit contains an antibacterial agent, which also prevents the growth of bacteria between the time of collection and the time of DNA purification. Immediately after collection, the samples were stored at −80°C to avoid bacterial growth. Previous studies have shown that buccal swabs contain around 11% human DNA, whereas saliva samples yield on average 68% human DNA [29, 30].

Both blood and saliva contain a variety of cell types, with different function and half-life, and presumably different susceptibility to external factors. DNA extracted from blood samples typically originates from leucocytes (granulocytes, lymphocytes, and monocytes), whereas human DNA from buccal swabs mainly stems from exfoliated epithelial cells. Human DNA from saliva on the other hand is derived from both leucocytes (granulocytes, lymphocytes, and monocytes) and exfoliated epithelial cells [31]. Blood, saliva, and buccal swabs differ not only in types of the cells they contain, but also in the viability of the cells. DNA from blood mostly stems from viable cells, whereas many cells in buccal swabs are dead (exfoliated cells). These differences might explain the differences in methylation pattern between the different tissues.

Our results indicated positive association for global DNA methylation and hydroxymethylation within the same tissue, that is, saliva. Interestingly, the direction of difference between global DNA methylation and hydroxymethylation levels in saliva and blood is opposite to each other, that is, low global DNA methylation and high global DNA hydroxymethylation in saliva compared to blood. This supports the hypothesis that 5hmC is involved as an intermediate in the active demethylation of 5mC. No association in methylation levels of blood and saliva LINE1 and Alu elements was observed with the DNA hydroxymethylation levels in blood and saliva DNA, respectively. Since LINE1 and Alu elements methylation does not represent the complete cellular pool of global DNA methylation, this might explain the lack of this association along with other factors, for example, small sample size. DNA methylation and hydroxymethylation are tightly regulated in live cells. Saliva comprises mainly dead cells, which can explain the low levels of global DNA methylation but high levels of global DNA hydroxymethylation (i.e., dead cells lose control in methylating the repetitive elements which would increase the global DNA hydroxymethylation) as compared to the blood, which contains the live cells tightly regulating their genome. The mechanisms explaining how this process is controlled and mediated in different cell types are still unclear [32].

## 5. Conclusion

In conclusion, both global DNA methylation and hydroxymethylation of cytosine can be quantified simultaneously by LC/MS-MS. Global DNA methylation and hydroxymethylation in saliva and blood DNA do not seem to be comparable and consequently saliva cannot be considered as a surrogate for blood for epigenetic endpoints. There are indications of a positive association between global DNA methylation and hydroxymethylation within the same tissue, that is, saliva.

## 6. Future Perspectives

Epigenomics is an active research field driven by the massive amount of new information. New cellular pathways are emerging as knowledge of epigenomics is growing. Recently, the identification and tissue-specific distribution of DNA hydroxymethylation lead to speculation that DNA hydroxymethylation is not just a passive mark but could play an important cellular function. Further research in tissue-specific correlation of different epigenetic factors will help understand how epigenetic factors play a role in regulating the activity of genes.

## Supplementary Material

The supplementary table 1 reports the individual methylation levels of Long interspersed nucleotide elements (LINE-1) and the AluSX (Alu) assessed using PCR-pyrosequencing of bisulfite-treated blood and saliva DNA. No significant correlation was observed between the methylation levels of blood and saliva Alu elements (spearman rho = 0.297, *P*= 0.303). Methylation levels of blood and saliva LINE1 elements were also not significantly correlated (spearman rho = -0.196, *P*= 0.503), although in general lower methylation levels were observed for LINE1 and Alu elements in saliva DNA compared to their methylation levels in blood DNA.

## Figures and Tables

**Table 1:** %methylation and %hydroxymethylation in blood and saliva.

	Mean	Standard deviation	Minimum	Maximum
Saliva DNA %hydroxymethylation	0.036	0.011	0.019	0.053
Blood DNA % hydroxymethylation	0.027	0.004	0.021	0.032
Saliva DNA %methylation	4.61	0.80	2.36	5.75
Blood DNA % methylation	5.70	0.22	5.25	6.12
