# Supplementary material for: Global Methylation and Hydroxymethylation in DNA from Blood and Saliva in Healthy Volunteers
Source: Biomed Res Int. 2015 May 18;2015:845041. doi: 10.1155/2015/845041 (PMC4450276; doi:10.1155/2015/845041)
Supplement: Supplementary file 1 — The supplementary table 1 reports the individual methylation levels of Long interspersed nucleotide elements (LINE-1) and the AluSX (Alu) assessed using PCR-pyrosequencing of bisulfite-treated blood and saliva DNA. No significant correlation was observed between the methylation levels of blood and saliva Alu elements (spearman rho = 0.297, P= 0.303). Methylation levels of blood and saliva LINE1 elements were also not significantly correlated (spearman rho = -0.196, P= 0.503), although in general lower methylation levels were observed for LINE1 and Alu elements in saliva DNA compared to their methylation levels in blood DNA. [file 845041.f1.docx]

Supplementary table 1: % Methylation levels of long interspersed nucleotide elements (LINE1) and Alu elements in blood and saliva assessed by PCR-Pyrosequencing.

| Subjects | LINE1 methylation (%) | | AluSX methylation (%) | |
| --- | --- | --- | --- | --- |
|  | Blood | Saliva | Blood | Saliva |
| 1 | 83.78 | 70.79 | 39.96 | 33.91 |
| 2 | 80.73 | 51.93 | 37.24 | 28.75 |
| 3 | 93.21 | 81.18 | 40.74 | 37.78 |
| 4 | 87 | 42.97 | 38.44 | 30.52 |
| 5 | 81.15 | 73.17 | 42.18 | 30.72 |
| 6 | 81.45 | 58.05 | 40.09 | 34.59 |
| 7 | 74.73 | 81.28 | 40.99 | 31.18 |
| 8 | 78.65 | 71.74 | 39.7 | 33.24 |
| 9 | 82.31 | 87.16 | 39.57 | 31.2 |
| 10 | 76.92 | 68.5 | 38.83 | 31.85 |
| 11 | 75.73 | 75.29 | 39.78 | 30.92 |
| 12 | 83.55 | 69.21 | 39.1 | 32.42 |
| 13 | 78.41 | 79.11 | 38.21 | 28.65 |
| 14 | 82.18 | 72.66 | 37.88 | 36.02 |
